# Supplementary material for: Causal interplay between lactose intolerance and gut microbiota: a combined bidirectional Mendelian randomization and in vivo validation study
Source: Front Nutr. 2026 Jun 1;13:1803337. doi: 10.3389/fnut.2026.1803337 (PMC13265576; doi:10.3389/fnut.2026.1803337)
Supplement: Supplementary file 1 [file Data_Sheet_1.zip › supplementary materials/Forward/leave one out cross validation/ebi-a-GCST90027836.finngen_R12_E4_LACTONAS.pdf]

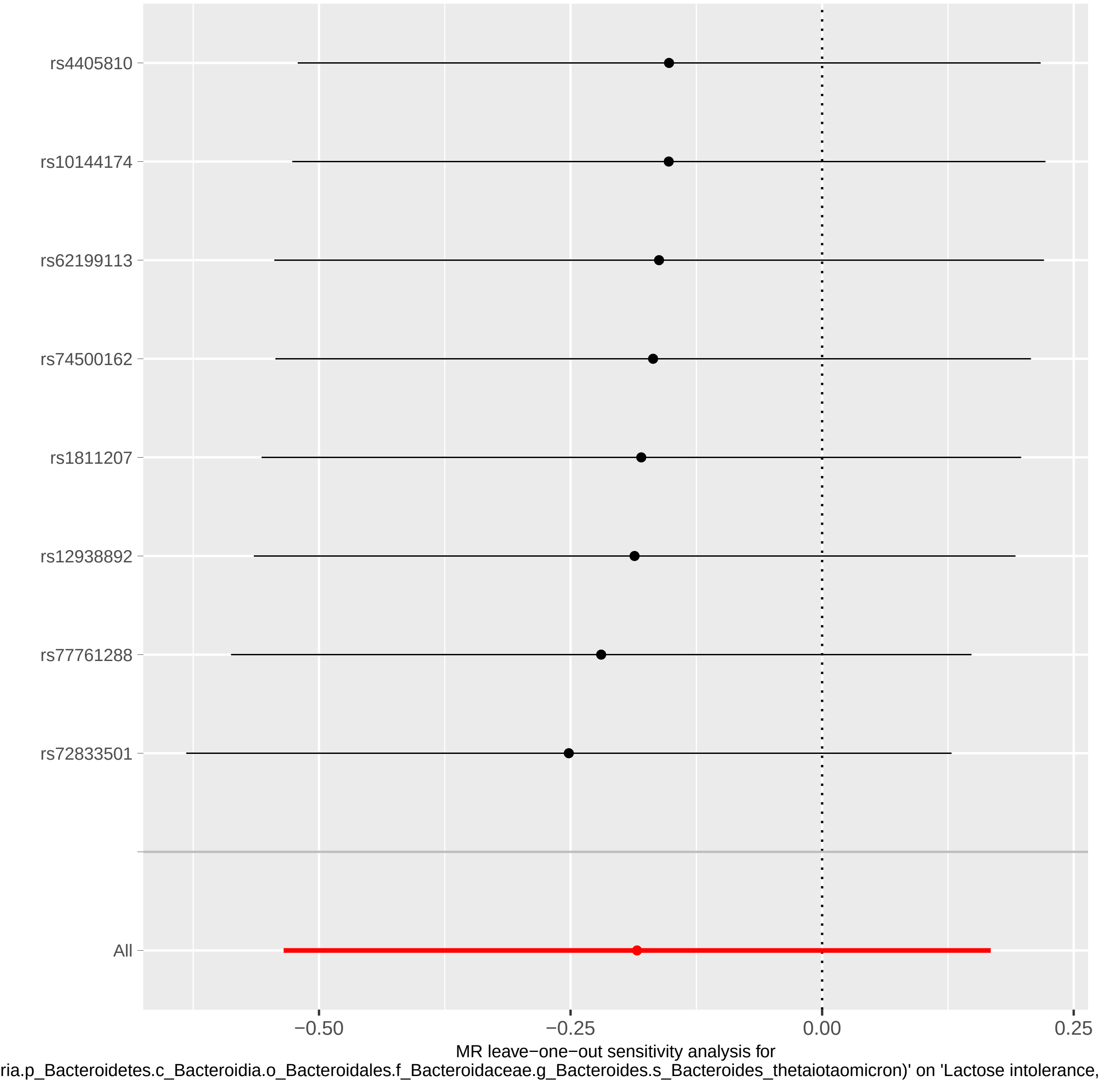
rs4405810
rs10144174
rs62199113
rs74500162
rs1811207
rs12938892
rs77761288
rs72833501
All
−0.50
−0.25
0.00
0.25
MR leave−one−out sensitivity analysis for
ria.p_Bacteroidetes.c_Bacteroidia.o_Bacteroidales.f_Bacteroidaceae.g_Bacteroides.s_Bacteroides_thetaiotaomicron)' on 'Lactose intolerance,
